# Opicapone as Add-On Therapy to Continuous Subcutaneous Foslevodopa/Foscarbidopa Infusion: Clinical Improvement and Wearable Sensor-Based Gait Analysis

**DOI:** 10.3390/brainsci16050545

**Published:** 2026-05-21

**Authors:** Paolo Solla, Carla Masala, Gianluca Martinez, Raffaele Galiero, Valentina Floris, Elisa Ogana, Valentina Canu, Francesco Loy, Tommaso Ercoli

**Affiliations:** 1 Neurology Unit, University Hospital of Sassari, Viale S. Pietro 10, 07100 Sassari, Italy; g.martinez10@phd.uniss.it (G.M.); raffaele_ga@outlook.it (R.G.); valentina.floris13@gmail.com (V.F.); elisaogana@gmail.com (E.O.); valcanu0500@gmail.com (V.C.); ercolitommaso@me.com (T.E.); 2 Department of Biomedical Sciences, University of Cagliari, SP 8 Cittadella Universitaria, 09042 Monserrato, Italy; cmasala@unica.it (C.M.); floy@unica.it (F.L.); 3 Department of Medical Sciences and Public Health, University of Cagliari, SP 8 Cittadella Universitaria, 09042 Monserrato, Italy

**Keywords:** Parkinson’s disease, opicapone, foslevodopa, foscarbidopa, wearable sensor monitoring

## Abstract

Background/Objectives: Continuous subcutaneous foslevodopa/foscarbidopa infusion (CSFLI) improves motor fluctuations in advanced Parkinson’s disease (PD), but some patients continue to experience residual motor and non-motor fluctuations despite optimized infusion parameters. We describe two patients receiving CSFLI in whom the addition or reintroduction of opicapone was associated with improvement in persistent fluctuations. In one patient, clinical findings were further explored using wearable sensor monitoring. Methods: Two patients with advanced PD treated with CSFLI and residual fluctuations received add-on opicapone. Clinical evaluation included neurological examination, assessment of motor fluctuations, and documentation of antiparkinsonian therapy before and after opicapone introduction. In one patient, motor performance was additionally evaluated with wearable sensor monitoring during the Timed Up and Go test and the 6-minute walk test before (T0) and three months after opicapone introduction (T1). Results: In both cases, opicapone 50 mg once daily was associated with rapid improvement in residual motor and non-motor fluctuations during CSFLI therapy. In the first patient, freezing episodes and unpredictable akinesia resolved. In the second patient, the reintroduction of opicapone improved morning slowness, axial symptoms, and dorsal pain. Wearable sensor analysis showed increased walking distance during the 6-minute walk test, higher walking speed, reduced Timed Up and Go duration, increased step length, and reduced step length variability. Conclusions: These preliminary observations suggest that opicapone may deserve further investigation as a potential adjunctive strategy in selected patients receiving CSFLI who continue to experience residual fluctuations despite optimized infusion therapy. Wearable sensor monitoring may provide objective support for treatment assessment in this setting.

## 1. Introduction

Advanced Parkinson’s disease (PD) is frequently complicated by disabling motor fluctuations, including both predictable and unpredictable OFF episodes, the freezing of gait, peak-dose dyskinesias, and non-motor fluctuations such as pain and axial symptoms, which severely impact quality of life despite optimized oral pharmacotherapy [1]. Device-aided therapies, including levodopa–carbidopa intestinal gel (LCIG) and continuous subcutaneous apomorphine or foslevodopa/foscarbidopa infusion, have been developed to provide continuous dopaminergic stimulation, improving motor symptoms and reducing levodopa-related motor complications [2].

Continuous subcutaneous foslevodopa/foscarbidopa infusion (CSFLI) delivers a water-soluble prodrug combination that is rapidly converted in the subcutaneous tissue to levodopa and carbidopa, achieving stable plasma levodopa concentrations comparable to those obtained with LCIG. The system allows programmable basal rates, adjustable high and low infusion rates, and optional extra doses to manage motor fluctuations [3,4]. However, as previously reported in LCIG cohorts, a substantial proportion of patients may continue to experience residual OFF time, particularly in the afternoon and evening, often requiring multiple extra doses per day [5]. In this context, combining infusion therapy with peripheral catechol-O-methyltransferase (COMT) inhibition represents a biologically plausible strategy to increase levodopa bioavailability and optimize dopaminergic stimulation [6].

Opicapone is a third-generation, long-acting, peripherally selective COMT inhibitor administered once daily as adjunctive therapy to levodopa/carbidopa in patients with PD and motor fluctuations [7]. Clinical trials in patients receiving oral levodopa have shown that opicapone increases levodopa exposure and significantly reduces OFF time, with a safety profile mainly characterized by dopaminergic adverse events, particularly dyskinesia [8].

Evidence on the use of opicapone in patients receiving infusion therapies remains limited. A single case report in a PD patient treated with LCIG suggested that opicapone improved clinical symptoms, reduced extra-dose requirements while increasing levodopa and reducing 3-O-methyldopa concentrations [9]. Furthermore, a retrospective series of 11 LCIG-treated patients reported that opicapone 50 mg once daily was associated with a median 24.8% reduction in daily LCIG volume without significant worsening of dyskinesia [10].

Despite these observations in LCIG-treated patients, evidence regarding the use of opicapone in combination with CSFLI is still limited [11]. Differently from previous LCIG-based reports, the present observations refer to CSFLI, a distinct and more recently introduced levodopa delivery system, and include wearable sensor-based assessment of gait and motor performance in one patient. In this report, we describe two illustrative cases in which the addition of opicapone to CSFLI was associated with improvement in residual motor and non-motor fluctuations.

## 2. Materials and Methods

This report describes two patients with advanced PD treated with CSFLI at the Movement Disorders Center of the University Hospital of Sassari. The cases were selected because both patients experienced clinically relevant residual motor and non-motor fluctuations despite optimized infusion therapy and subsequently received opicapone as an add-on strategy, with available clinical follow-up. In one patient, wearable sensor-based motor assessment was also available. The study was conducted in accordance with the principles of the Declaration of Helsinki. Written informed consent for the use of clinical data for research purposes was obtained from both patients (Ethics Committee of Sardinia—protocol n. 448/2025).

Clinical evaluation included neurological examination, assessment of motor fluctuations, and documentation of antiparkinsonian treatment before and after the introduction of opicapone. Cognitive function was assessed using the Montreal Cognitive Assessment (MoCA), and disease severity was staged according to the Hoehn and Yahr scale and the MDS-UPDRS part III. Motor performance was further explored in one patient using wearable sensor monitoring to obtain an objective assessment of gait and mobility changes associated with treatment.

### Wearable Sensor Assessment

Motor performance was assessed using a wearable sensor system (Dynaport MoveMonitor, McRoberts BV, Hague, The Netherlands). The device is a lightweight inertial sensor worn at the level of the lower back (lumbar region) and allows the continuous monitoring of mobility-related parameters during daily activities. Motor function was evaluated through outpatient motor tests and home monitoring. Clinical assessments included the Timed Up and Go (TUG) test and the 6-minute walk test (6MWT). For the TUG test, five trials were performed at each time point, and the average value was used for analysis. During each trial, the patient was instructed to stand up from a chair, walk three meters, turn around, walk back, and sit down again. The 6MWT was performed in the same location at both assessments, using the same 25 m corridor. The patient was instructed to walk as far as possible within six minutes and was informed of the remaining time every minute.

Evaluations were performed before the introduction of opicapone (T0) and after three months of follow-up (T1). Both assessments were conducted at the same time of day, in the same medication state, and by the same evaluator. The same software version and data-processing algorithms were used for both recordings. The Dynaport system provides several gait-related parameters derived from trunk acceleration signals, including step length, walking speed, cadence, and distance covered.

## 3. Results

### 3.1. Case 1

A 70-year-old man with a 20-year history of PD, which started at age 50 with left-hand tremor, was admitted for treatment optimization due to advanced disease with severe motor complications. At baseline, he experienced frequent and severe motor fluctuations with both predictable and unpredictable OFF episodes, as well as disabling peak-dose dyskinesias during ON periods. At admission, antiparkinsonian treatment included levodopa/benserazide 200/50 mg (one tablet at 08:00 and half a tablet at 12:00, 16:00, and 20:00), prolonged-release ropinirole 4 mg twice daily, safinamide 100 mg once daily, and controlled-release levodopa/carbidopa 100/25 mg at bedtime. Neurological examination showed a left-dominant Parkinsonian syndrome with Pisa syndrome, cervical dystonia, and right-sided dyskinesias in the ON state. At baseline during the ON phase, the MoCA score was 22/30, the Hoehn and Yahr stage was 2, and the MDS-UPDRS part III was 45.

CSFLI was started after patient and caregiver education, with oral levodopa discontinued and initial parameters set at a base rate of 0.17 mL/h, a high rate of 0.19 mL/h, and a low rate of 0.15 mL/h. Conversion from oral levodopa to CSFLI was performed according to the product prescribing information. The patient initially showed a significant improvement in motor fluctuations and dyskinesias, with improvements in trunk posture and cervical dystonia.

However, over the next four weeks, he developed recurrent and disabling daytime fluctuations characterized by prolonged freezing episodes and unpredictable early-morning and evening akinesia, despite gradually increasing the infusion up to a base rate of 0.19 mL/h and a high rate of 0.21 mL/h. These motor blocks were not responsive to extra doses of subcutaneous levodopa, with the patient requiring an average of three extra doses per day. Given the persistence of these disabling fluctuations, opicapone 50 mg once daily at bedtime was introduced. This adjustment led to an almost complete resolution of motor fluctuations and freezing episodes. Infusion rates were not further modified after opicapone introduction. No adverse events were reported, including neuropsychiatric symptoms, worsening dyskinesia, or cutaneous complications. At four-month follow-up, the clinical benefit persisted without the need for further adjustments of the infusion therapy.

### 3.2. Case 2

An 80-year-old man with an eight-year history of PD presented with a predominantly bilateral, rigid–akinetic phenotype, more pronounced on the right side. His disease course was complicated by evening OFF periods characterized by severe bradykinesia and rigidity, marked forward trunk flexion with camptocormia, and prominent non-motor fluctuations, including severe dorsal pain, particularly in the evening, and poorly responsive to oral therapy. At admission, antiparkinsonian treatment included levodopa/benserazide 200/50 mg (half a tablet four times daily), safinamide 100 mg once daily, opicapone 50 mg once daily at night, and prolonged release ropinirole 6 mg in the morning. Neurological examination showed a typical bilateral rigid–akinetic Parkinsonian syndrome with right-side predominance, camptocormia, and rightward trunk flexion, without clear dyskinesias. During the ON phase at baseline, the Hoehn and Yahr stage was 2.5, the MDS-UPDRS part III was 29, and the MoCA score was 19/30.

CSFLI was initiated after appropriate patient and caregiver education. The initial parameters were a base rate of 0.18 mL/h, a high rate of 0.19 mL/h, and a low rate of 0.15 mL/h. Oral levodopa and opicapone were discontinued according to the prescribing information for foslevodopa/foscarbidopa. Clinically, the patient showed significant improvement, with reductions in OFF time, axial symptoms, and dorsal pain.

Over the following three months, the patient reported persistence of overall benefit but complained of increased morning slowness, partial recurrence of trunk flexion, and the re-emergence of dorsal pain in the early hours. The infusion was therefore increased to a base rate of 0.19 mL/h and a high rate of 0.20 mL/h, while the low rate remained unchanged.

At subsequent follow-up, motor and non-motor fluctuations persisted. At that time, he was using an average of three extra doses per day, without clear clinical benefit. Opicapone 50 mg once daily was therefore reintroduced in the late evening. Within two weeks, the patient reported complete resolution of symptoms, with optimal control of both motor and non-motor fluctuations. Infusion rates were not further modified after opicapone reintroduction. No adverse events were reported after opicapone reintroduction, including neuropsychiatric symptoms, worsening dyskinesia, or cutaneous complications. At approximately five months of follow-up, he maintained stable and satisfactory clinical control without further adjustments of the infusion therapy.

**Figure 1:**
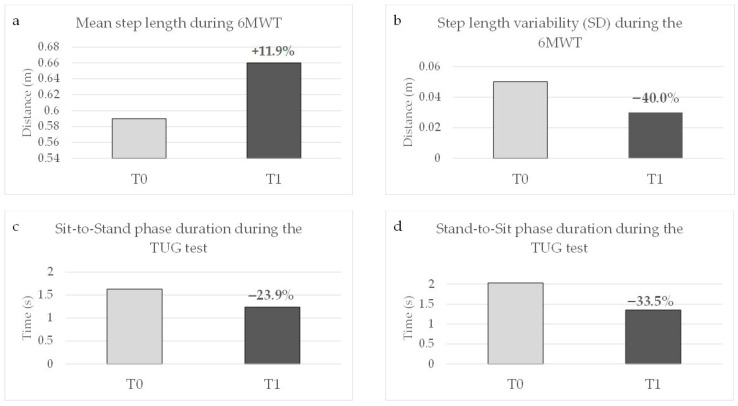
Comparison between baseline (T0) and post-intervention (T1) for all outcomes. (**a**) Mean step length during the 6-minute walk test (6MWT). (**b**) Step length variability during the 6MWT. (**c**) Sit-to-Stand phase duration during the Timed Up and Go (TUG) test. (**d**) Stand-to-Sit phase duration during TUG test.

In this patient, motor performance was further explored using wearable sensor monitoring (Dynaport MoveMonitor, McRoberts BV, The Netherlands). Assessments included the 6MWT and the TUG test, performed before the introduction of opicapone (T0) and after three months of follow-up (T1). Wearable sensor monitoring provided supportive objective information consistent with the clinical improvement observed after the introduction of opicapone. During the 6MWT, total walking distance increased from 415.91 m to 455.73 m (+9.6%), while mean walking speed increased from 1.25 m/s to 1.40 m/s (+12.0%). Performance in the TUG test also improved, with a reduction in total test duration from 9.32 ± 1.71 s to 7.67 ± 0.34 s (−17.7%). Consistent with these findings, gait parameters derived from wearable sensor monitoring showed an increase in mean step length during the 6MWT (+11.9%) and a reduction in step length variability (−40%). Improvements were also observed in postural transition phases during the TUG test, with shorter sit-to-stand (−23.9%) and stand-to-sit (−33.5%) phase durations (Figure 1).

## 4. Discussion

In this report, we describe two patients with advanced PD treated with CSFLI who experienced persistent motor and non-motor fluctuations despite apparently optimized infusion parameters. In both cases, the addition or reintroduction of opicapone was associated with the improvement of symptoms that persisted during follow-up, including resolution of freezing episodes and early-morning akinesia in one patient and improvement in motor and non-motor fluctuations in the other. These preliminary observations suggest that COMT inhibition may deserve further investigation as a potential adjunctive approach in selected patients receiving CSFLI who continue to experience residual motor and non-motor fluctuations despite optimized infusion parameters.

CSFLI is an effective device-aided therapy for the management of motor fluctuations in advanced PD [12,13,14]. By providing continuous dopaminergic stimulation, this approach reduces the pulsatile stimulation associated with oral levodopa and improves motor control [14]. However, despite careful titration of infusion parameters, a proportion of patients may continue to experience residual or re-emergent fluctuations, particularly during the early morning or evening hours. In clinical practice, addressing these fluctuations by progressively increasing infusion rates may not always be effective and may expose patients to higher cumulative levodopa doses [15,16,17].

Evidence on the use of opicapone in patients receiving infusion therapies remains limited [18]. Previous reports in patients treated with LCIG have suggested that COMT inhibition may reduce infusion requirements and improve motor fluctuations without worsening dyskinesia [9,10]. Our observations extend these findings to patients treated with CSFLI, suggesting that a similar pharmacological interaction may occur in this therapeutic context.

According to the prescribing information for foslevodopa/foscarbidopa, oral levodopa therapy should be converted to infusion therapy based on the calculation of levodopa equivalents. When patients are receiving COMT inhibitors, the prescribing information recommends applying a correction factor of 1.33 to the calculated levodopa equivalent dose, reflecting the increase in levodopa bioavailability associated with COMT inhibition. In clinical practice, COMT inhibitors are therefore commonly discontinued when initiating CSFLI, as continuous levodopa delivery is expected to provide sufficient pharmacokinetic stability.

A possible pharmacological explanation for our observations relates to the effect of COMT inhibition on levodopa metabolism [19,20]. Peripheral COMT activity converts levodopa into 3-O-methyldopa, which may compete with levodopa for transport across the blood–brain barrier [19,20]. Therefore, reduced 3-O-methyldopa formation could theoretically improve levodopa availability to the central nervous system, even during continuous levodopa infusion. In addition, previous studies have linked long-term levodopa metabolism, homocysteine elevation, B-vitamin depletion, and peripheral neuropathy in patients receiving infusion therapies [19,20]. However, these biochemical parameters were not assessed in our patients; therefore, this mechanism should be interpreted as a pharmacological rationale supported by previous literature rather than as direct evidence from the present cases [19,20].

In one patient, the clinical improvement observed after the reintroduction of opicapone was further supported by wearable sensor monitoring. Objective gait analysis showed improvements in walking distance, gait speed, step length, and gait variability during the 6MWT, as well as faster postural transitions during the TUG test. Although these observations are limited to a single case, they provide objective support to the clinical impression of improved motor performance and highlight the potential value of wearable technologies in the evaluation of treatment response in advanced PD. Such technologies may offer a valuable tool for the quantitative assessment of treatment response in advanced PD, particularly in patients receiving device-aided therapies [21].

This report has several important limitations. First, the very small number of patients and the observational nature of the study do not allow generalizable conclusions regarding treatment efficacy. Given the observational nature of the report, a causal effect of opicapone cannot be established, and other factors, including clinical variability and treatment optimization over time, may have contributed to the observed improvement. In addition, axial and pain-related manifestations such as Pisa syndrome, cervical dystonia, camptocormia, and dorsal pain may have multifactorial mechanisms and should not be attributed exclusively to dopaminergic changes. Nevertheless, improvement in Pisa syndrome following continuous subcutaneous foslevodopa/foscarbidopa infusion has recently been described, suggesting that axial symptoms may improve in selected patients receiving continuous dopaminergic stimulation [22]. Second, wearable sensor monitoring was available in only one patient and should therefore be interpreted as supportive objective information rather than confirmatory evidence. Third, although baseline motor status was characterized using the MDS-UPDRS part III, standardized longitudinal motor scale assessments were not available to quantify the clinical response after opicapone introduction. Therefore, these findings should be interpreted as preliminary clinical observations and require confirmation in larger prospective studies.

## 5. Conclusions

In conclusion, these preliminary observations suggest that opicapone may deserve further investigation as a potential adjunctive treatment in selected patients receiving CSFLI who continue to experience residual motor and non-motor fluctuations despite optimized infusion parameters. Larger prospective studies with standardized clinical scales and objective motor assessments are needed to confirm these findings.

## Data Availability

The datasets generated and analyzed during the current study are available from the corresponding author on reasonable request due to privacy.

## Abbreviations

The following abbreviations are used in this manuscript:

CSFLI	Continuous subcutaneous foslevodopa/foscarbidopa infusion
PD	Parkinson’s disease
COMT	catechol-O-methyltransferase
LCIG	levodopa–carbidopa intestinal gel
MoCA	Montreal Cognitive Assessment
TUG	Timed Up and Go
6MWT	6-minute walk test

## References

[1] Regensburger M., Csoti I., Jost W.H., Kohl Z., Lorenzl S., Pedrosa D.J., Lingor P. (2026). Motor and non-motor fluctuations in Parkinson’s disease: The knowns and unknowns of current therapeutic approaches. J. Neural. Transm..

[2] Pirtošek Z., Leta V., Jenner P., Vérin M. (2023). Should continuous dopaminergic stimulation be a standard of care in advanced Parkinson’s disease?. J. Neural. Transm..

[3] Soileau M.J., Aldred J., Budur K., Fisseha N., Fung V.S., Jeong A., Kimber T.E., Klos K., Litvan I., O’Neill D. (2022). Safety and efficacy of continuous subcutaneous foslevodopa-foscarbidopa in patients with advanced Parkinson’s disease: A randomised, double-blind, active-controlled, phase 3 trial. Lancet Neurol..

[4] Rosebraugh M., Stodtmann S., Liu W., Facheris M.F. (2022). Foslevodopa/foscarbidopa subcutaneous infusion maintains equivalent levodopa exposure to levodopa-carbidopa intestinal gel delivered to the jejunum. Park. Relat. Disord..

[5] Thomas I., Memedi M., Westin J., Nyholm D. (2019). The effect of continuous levodopa treatment during the afternoon hours. Acta Neurol. Scand..

[6] Jenner P., Nyholm D. (2026). COMT inhibition with entacapone for patients with Parkinson’s disease and motor complications: The novelty of continuous infusion. J. Neural. Transm..

[7] Greenwood J., Pham H., Rey J. (2020). Opicapone: A third generation COMT inhibitor. Clin. Park. Relat. Disord..

[8] Ferreira J.J., Lees A., Rocha J.F., Poewe W., Rascol O., Soares-da-Silva P. (2019). Long-term efficacy of opicapone in fluctuating Parkinson’s disease patients: A pooled analysis of data from two phase 3 clinical trials and their open-label extensions. Eur. J. Neurol..

[9] Miyaue N., Ito Y.H., Ochi C., Yamanishi Y., Tada S., Ando R., Nagai M. (2023). Impact of concomitant use of opicapone during levodopa-carbidopa intestinal gel treatment. J. Neurol. Sci..

[10] Leta V., van Wamelen D.J., Sauerbier A., Jones S., Parry M., Rizos A., Chaudhuri K.R. (2020). Opicapone and Levodopa-Carbidopa Intestinal Gel Infusion: The Way Forward Towards Cost Savings for Healthcare Systems?. J. Park. Dis..

[11] Koeglsperger T., Berberovic E., Dresel C., Haferkamp S., Kassubek J., Müller R., Oehlwein C., Paus S., Urban P.P. (2026). Real-world experience with continuous subcutaneous foslevodopa/foscarbidopa infusion: Insights and recommendations. J. Neural. Transm..

[12] Fung V.S.C., Aldred J., Arroyo M.P., Bergquist F., Boon A.J.W., Bouchard M., Bray S., Dhanani S., Facheris M.F., Fisseha N. (2024). Continuous subcutaneous foslevodopa/foscarbidopa infusion for the treatment of motor fluctuations in Parkinson’s disease: Considerations for initiation and maintenance. Clin. Park. Relat. Disord..

[13] Aldred J., Freire-Alvarez E., Amelin A.V., Antonini A., Bergmans B., Bergquist F., Bouchard M., Budur K., Carroll C., Chaudhuri K.R. (2023). Continuous subcutaneous foslevodopa/foscarbidopa in Parkinson’s Disease: Safety and efficacy results from a 12-month, single-arm, open-label, phase 3 study. Neurol. Ther..

[14] Rosebraugh M., Liu W., Neenan M., Facheris M.F. (2021). Foslevodopa/Foscarbidopa is well tolerated and maintains stable Levodopa and Carbidopa exposure following subcutaneous infusion. J. Park. Dis..

[15] Pinna B., Fenu G., Mellino P., Serra G., Cadeddu G., Murgia D., Gozzi A., Antenucci P., Tessitore A., Ciaramaglia O. (2025). Titration Dynamics and Early Treatment Burden in Advanced Parkinson’s Disease: A Multicenter Comparison of CSFLI and LCIG Infusion Therapies. Mov. Disord. Clin. Pract..

[16] Brohée S., Roze E., Grabli D., Letrillart H., Mantisi L., Foucard C., Hainque E., Cormier F., Méneret A., Hauw F. (2025). Cognitive and Psychiatric Adverse Effects of Foslevodopa/Foscarbidopa in Patients with Parkinson’s Disease. Mov. Disord. Clin. Pract..

[17] Solla P., Velucci V., Defazio G., Ercoli T. (2025). Navigating the Neuropsychiatric Risks of Foslevodopa/Foscarbidopa in Patients with Parkinson’s Disease. Mov. Disord. Clin. Pract..

[18] Kwak N., Park J., Kang H.Y., Lee M.J., Suh J.K., Lee H. (2022). Efficacy and Safety of Opicapone for Motor Fluctuations as an Adjuvant to Levodopa Therapy in Patients with Parkinson’s Disease: A Systematic Review and Meta-Analysis. J. Park. Dis..

[19] Cossu G., Ceravolo R., Zibetti M., Arca R., Ricchi V., Paribello A., Murgia D., Merola A., Romagnolo A., Nicoletti V. (2016). Levodopa and neuropathy risk in patients with Parkinson disease: Effect of COMT inhibition. Park. Relat. Disord..

[20] Pauls K.A.M., Toppila J., Koivu M., Eerola-Rautio J., Udd M., Pekkonen E. (2021). Polyneuropathy monitoring in Parkinson’s disease patients treated with levodopa/carbidopa intestinal gel. Brain Behav..

[21] Moreau C., Rouaud T., Grabli D., Benatru I., Remy P., Marques A.R., Drapier S., Mariani L.L., Roze E., Devos D. (2023). Overview on wearable sensors for the management of Parkinson’s disease. npj Park. Dis..

[22] Ercoli T., Velucci V., Defazio G., Solla P. (2026). Improvement of Pisa syndrome in Parkinson’s disease following continuous subcutaneous infusion of foslevodopa/foscarbidopa. Neurol. Sci..

